# Obstructive sleep apnea and its association with coronary artery calcium volume in asymptomatic male subjects

**DOI:** 10.1038/s41598-023-47666-9

**Published:** 2023-12-07

**Authors:** Min Young Seo, Seung Hoon Lee, Hyo Yeol Kim

**Affiliations:** 1 Department of Otorhinolaryngology - Head and Neck Surgery, Korea University College of Medicine, Korea University Ansan Hospital, Ansan, South Korea; 2 Department of Otorhinolaryngology - Head and Neck Surgery, Samsung Medical Center, Sungkyunkwan University School of Medicine, 81 Irwon-Ro, Gangnam-Gu, Seoul, 06351 Korea

**Keywords:** Cardiology, Medical research

## Abstract

The aim of this study was to analyze the association between various parameters related to obstructive sleep apnea (OSA) and coronary artery calcium (CAC) volume. We retrospectively reviewed the medical records of 315 male subjects who underwent standard polysomnography (PSG) and coronary artery computed tomography. In this study, we found that only the apnea index (AI) and minimal oxygen saturation (minimal SaO_2_) were independently associated with CAC volume after adjustment for confounders; for a 1/h increase in the AI, the CAC volume increased by 1.311 mm^3^, and for a 1% increase in the minimal SaO_2_, the CAC volume decreased by 2.187 mm^3^. We also found that the CAC volume was significantly different between the habitual snorer and the severe OSA group (21.27 ± 40.79 vs 71.33 ± 175.00, *p* = 0.042). Moreover, the CAC volume was significantly different between the first and fourth quartile groups of the AI (32.42 ± 59.54% vs. 78.74 ± 198.50, *p* = 0.048), but not among groups according to the hypopnea index quartile. Therefore, we concluded that among various OSA-related PSG parameters, the AI and minimal SaO_2_ was independently associated with the CAC volume and significantly related to upcoming cardiovascular events in middle-aged men.

## Introduction

Obstructive sleep apnea (OSA) is a common disorder and males are known to be more susceptible it; its estimated prevalence is approximately 22% in men and 17% in women^1^. In the Medical Subject Headings terms, OSA is defined as a disorder characterized by recurrent apneas during sleep despite persistent respiratory efforts due to upper airway obstruction, which induces hypoxia and hypercapnia^2^. These events are associated with various clinical morbidities, including cardiovascular disease (CVD), with sympathetic activation during sleep^3,4^. Coronary artery calcium (CAC) reflects the subclinical status of coronary artery disease and predicts cardiovascular events^5^. Previously, we have shown the association between the CAC score using the Agatston scoring method and various respiratory parameters associated with OSA, and reported that the factors that reflect nocturnal hypoxia are significantly associated with the current status of the CAC score and its progression over time^6,7^. Although the Agatston scoring method is the standard modality for quantifying CAC^8^, Criqui et al.^9^ reported that the CAC volume was positively associated with CVD events in the Multi-Ethnic Study of Atherosclerosis (MESA). Moreover, they also reported that most CVD risk factors were significantly associated with higher CAC volume scores. To date, several studies have reported the association between CAC and OSA^6,10–12^; however, no study has used the CAC volume. Therefore, we performed the present study to analyze the association between various parameters associated with OSA and the CAC volume (mm^3^) in middle-aged male subjects who snore and absence of symptoms related to cardiovascular conditions.

## Material and methods

### Study participants

We retrospectively reviewed the medical records of 315 male subjects who underwent both overnight standard polysomnography (PSG) and coronary artery computed tomography (CT) between January 2003 and December 2016 for a general health check-up. Coronary artery CT scan was conducted within 12 months of PSG investigation. None of the participants had any cardiovascular symptoms or a history of CVD. Moreover, no participants received any treatment for OSA, such as upper airway surgery, positive airway pressure therapy, or oral appliance use.

### Polysomnographic evaluation

Overnight standard PSG was performed using an Alice 3 device (Healthdyne Technologies®, Marietta, GA, USA) or Somnologica Studio (Embla Systems, Broomfield, CO, USA) at sleep study center of our tertiary hospital with observation by well-trained sleep physician according to the following American Academy of Sleep Medicine (AASM) recommended neurophysiologic and respiratory signals: Electroencephalography, electromyography, electro-oculography, and electrocardiography. Airflow was monitored using an oro-nasal thermistor and nasal pressure transducer for the detection of apnea and hypopnea, respectively. Chest and abdominal wall movements were detected using plethysmography to determine respiratory efforts. Oxygen saturation was recorded using finger-pulse oximetry. Overnight standard PSG data were interpreted manually by a well-trained sleep technician and confirmed by a certified sleep physician according to the AASM-recommended guidelines^13^. Apnea was defined as a reduction of oro-nasal airflow by > 90%, and hypopnea was defined as a decrease in nasal airflow of ≥ 30% with ≥ 4% desaturation or a reduction in airflow ≥ 50% with ≥ 3% desaturation for more than 10 s, from pre-event baseline or associated with arousal. The severity of OSA was defined according to the apnea–hypopnea index (AHI) as habitual snorer (HS, AHI < 5/h) and mild (5 ≤ AHI < 15/h with associated clinical features), moderate (15 ≤ AHI < 30/h), and severe OSA (30/h ≤ AHI)^14^.

### Evaluation of study participant characteristics and CVD risk factors

Demographic data of the study participants, such as age, sex, smoking, and body mass index (BMI; kg/m^2^) were measured on the day of PSG evaluation. The selection of optimal and suboptimal CVD risk factors for the analysis was carried out with reference to The Multi-Ethnic Study of Atherosclerosis^15^. The presence of hypertension (HTN) was defined as a systolic blood pressure of ≥ 140 mmHg, a diastolic blood pressure of ≥ 90 mmHg, or current use of antihypertensive medication. In addition, the presence of diabetes mellitus (DM) was defined as a fasting blood glucose of ≥ 126 mg/dl or current use of an oral hypoglycemic agent or insulin, and hypercholesterolemia was defined as a total cholesterol of ≥ 240 mg/dl or current use of lipid-lowering agents.

### Evaluation of the CAC volume

Forty-slice multi-detector CT (MDCT) (Brilliance-40; Philips, Cleveland, OH, USA) or 64-slice MDCT (LightSpeed VCT or LightSpeed HDI 750; GE Medical Systems, Milwaukee, WI, USA) was used to evaluate the CAC volume. The CAC volume score was measured by a single radiologist who specialized in cardiovascular imaging using previously published methods^16^.

### Statistical analysis

Statistical analysis was performed using the Statistical Package for the Social Sciences (SPSS, version 21, IBM Corporation; Armonk, NY, USA) and GraphPad Prism (version 7.0; San Diego, CA, USA). One-way analysis of variance (ANOVA) or Kruskal–Wallis test was performed on the basis of the data distribution to compare the demographics of the patients and evaluate the CAC volume as OSA severity. A linear by linear analysis was used to evaluate the categorical variables. For evaluating the association between various OSA-related parameters and the CAC volume, we performed a univariate linear regression analysis; a multivariate linear regression analysis was then conducted for parameters that were statistically significant with adjustment for age, BMI, DM, HTN, hyperlipidemia, and smoking status. A *p* value < 0.05 was considered statistically significant.

### Institutional review board statement

The study was conducted according to the guidelines of the Declaration of Helsinki, and approved by the Institutional Review Board of our tertiary Hospital (2021AS0207).

### Informed consent

Informed consent was waived because of the retrospective nature of the study and the analysis used anonymous clinical data and approved by the Institutional Review Board of Korea University Ansan Hospital (2021AS0207).

## Results

Among the 315 study participants, 39, 84, 86, and 106 subjects were regarded as belonging to HS, mild OSA, moderate OSA, and severe OSA groups, respectively. The patients’ demographic data according to OSA severity showed that the BMI significantly increased as the severity of OSA increased. In addition, the proportion of patients with DM, HTN, or hyperlipidemia was significantly higher in the moderate-to-severe OSA group than in the other groups. Various respiratory parameters such as minimal SaO_2_, apnea index (AI), hypopnea index (HI), AHI, respiratory disturbance index (RDI), and arousal index were significantly increased according to the severity of OSA, as expected. We also found that the proportion of deep sleep significantly decreased as the severity of OSA increased. The demographic data of the study participants are shown in Table 1. According to the results of the univariate analysis of demographics and various OSA-related parameters, age, BMI, minimal SaO_2_, AI, AHI, RDI, and arousal index were significantly associated with the CAC volume. As a result of the multivariate analysis for statistically significant parameters with adjustment for confounders (age, BMI, DM, HTN, hyperlipidemia, and smoking status), we found that the AI and minimal SaO_2_ were significantly associated with the CAC volume. Moreover, we found that age and BMI were significantly associated with confounders. According to the interpretation of the multivariate analysis, for a 1/h increase in the AI, the CAC volume increased by 1.311 mm^3^ and a 1% increase in the minimal SaO_2_, the CAC volume decreased by 2.187 mm^3^ (Table 2).

**Table 1:** Demographics of study subjects according to OSA severity.

Variables	Habitual snorer (AHI < 5, n = 39)	Mild OSA (5 ≤ AHI < 15, n = 84)	Moderate OSA (15 ≤ AHI < 30, n = 86)	Severe OSA (AHI ≥ 30, n = 106)	*P* value
Age (year)	52.28 ± 4.28 52 (49–55)	53.29 ± 4.32 53 (50–57)	53.30 ± 3.89 54 (50–56)	53.08 ± 3.87 53 (50–56)	0.579^a^
BMI (kg/m^2^)	24.21 ± 2.18 24.68 (22.28–27.23)	24.63 ± 2.16 24.44 (23.12–27.69)	25.74 ± 2.42 25.51 (23.89–27.26)	27.10 ± 3.32 26.87 (25.18–28.77)	< 0.001^a^
DM	2 (5.1%)	10 (11.9%)	9 (10.5%)	22 (20.8%)	0.014^b^
HTN	12 (30.8%)	29 (34.5%)	42 (48.8%)	46 (43.4%)	0.086^b^
Hyperlipidemia	20 (51.3%)	38 (45.2%)	57 (66.3%)	68 (64.2%)	0.013^b^
Smoke					
None	8 (20.5%)	28 (33.3%)	26 (30.3%)	28 (26.4%)	0.191^b^
Former	6 (15.4%)	20 (23.8%)	29 (33.7%)	36 (34%)	
Current	25 (64.1%)	36 (42.9%)	31 (36.0%)	42 (39.6%)	
Minimal SaO_2_ (%)	90.38 ± 3.24 91 (88–93)	86.94 ± 4.24 88 (85–90)	83.08 ± 4.93 83.5 (80–86)	78.25 ± 6.26 79 (74–84)	< 0.001^a^
AI (/h)	0.53 ± 0.87 0.2 (0–0.6)	1.98 ± 2.21 0.95 (0.43–2.8)	5.57 ± 5.42 4.2 (1.3–8.88)	25.77 ± 20.03 22.2 (9.2–34.43)	< 0.001^a^
HI (/h)	2.20 ± 1.29 2.2 (0.9–3.2)	8.03 ± 3.02 8.1 (6.1–10.1)	16.42 ± 5.22 16.15 (13.68–20.33)	24.41 ± 12.46 23.9 (15.13–32.28)	< 0.001^c^
AHI (/h)	2.73 ± 1.34 2.8 (1.6–4.1)	10.01 ± 2.80 9.8 (7.88–12.63)	21.99 ± 4.46 21.55 (18.10–25.70)	50.18 ± 16.62 45.75 (36.93–59.18)	< 0.001^a^
RDI (/h)	6.89 ± 5.35 5.00 (3.20–9.40)	14.33 ± 5.92 13.05 (10.13–16.00)	25.52 ± 5.01 25.15 (21.98–29.05)	52.80 ± 16.00 49.15 (40.68–61.18)	< 0.001^a^
Arousal index (/h)	1.93 ± 1.27 1.6 (1.1–2.6)	7.33 ± 5.19 6.65 (4.40–8.53)	14.70 ± 5.64 14.90 (9.98–18.00)	35.81 ± 17.64 31.60 (23.15–45.28)	< 0.001^a^
Deep sleep/TST (%)	4.99 ± 4.89 3.1 (1.4–9.2)	3.08 ± 4.36 1.2 (0.3–4.8)	2.41 ± 3.31 0.95 (0–3.63)	1.41 ± 2.57 0.20 (0–1.80)	< 0.001^a^

*BMI* body mass index, *DM* diabetes mellitus, *HTN* hypertension, *AI* apnea index, *HI* hypopnea index, *AHI* apnea–hypopnea index, *RDI* respiratory disturbance index, *TST* total sleep time.

Demographic characteristics are presented as mean ± SD, median (Q1–Q3) or as numbers (percentages).

^a^Kruskal–Wallis test.

^b^Linear by linear analysis.

^c^One-way analysis of variance (ANOVA).

**Table 2:** Association of between CAC volume and various OSA related parameters.

Variables	Model 1^a^		Model 2^b^	
	Univariate (r)	*P* value	Multivariate (β)	*P* value
Age	5.998	0.001		
BMI	7.045	0.008		
Minimal SaO_2_	− 2.405	0.035	− 2.187	0.030
AI	1.164	0.013	1.311	0.033
HI	0.528	0.436		
AHI	0.845	0.019		
RDI	0.855	0.021		
Arousal index	1.011	0.023		
Deep sleep/TST (%)	− 3.530	0.112		

Linear regression analysis.

*BMI* body mass index, *DM* diabetes mellitus, *HTN* hypertension, *AI* apnea index, *HI* hypopnea index, *AHI* apnea–hypopnea index, *RDI* respiratory disturbance index, *TST* total sleep time.

^a^Model 1: unadjusted model.

^b^Model 2: adjusted for age, BMI, DM, HTN, hyperlipidemia, smoking status.

**Figure 1:**
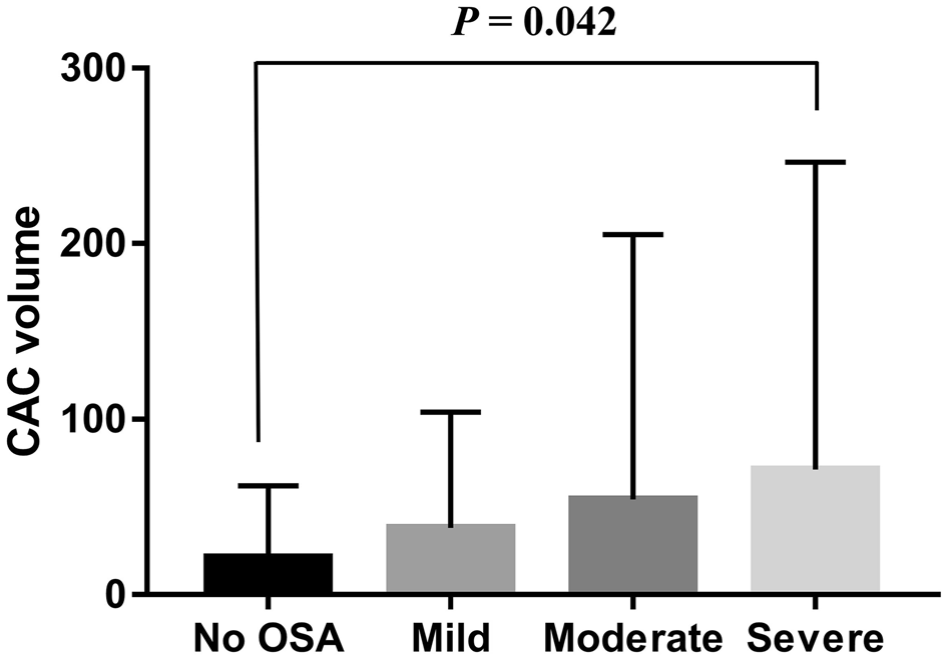
CAC volume difference between groups according to OSA severity. *CAC* coronary artery calcium, *OSA* obstructive sleep apnea, *HS* habitual snorer. One-way ANOVA with Bonferroni correction.

**Figure 2:**
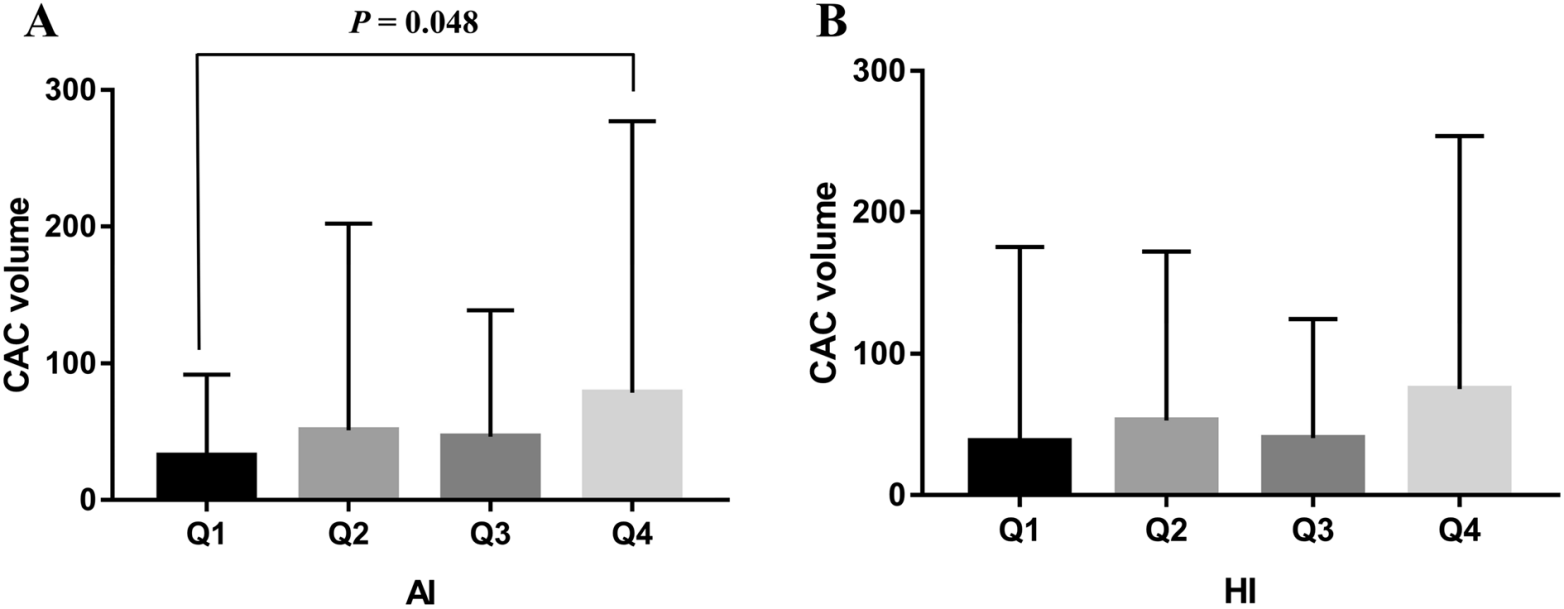
CAC volume difference between groups according to quartile of AI (**A**) and HI (**B**). *CAC* coronary artery calcium, *AI* apnea index, *HI* hypopnea index. One-way ANOVA with Bonferroni correction.

When comparing the CAC volume difference between groups according to OSA severity, we found that the CAC volume was significantly different between the HS and severe OSA groups (21.27 ± 40.79 vs. 71.33 ± 175.00, *p* = 0.042; data are presented as mean ± standard deviation [SD]) (Fig. 1). As mentioned above, among the various OSA-related parameters, the AI was the only one that was significantly associated with the CAC volume. Therefore, we performed a further assessment comparing the CAC volume among groups comprising the AI or HI quartiles of our study participants. The results showed that the CAC volume was also significantly different between the first quartile (Q1) and fourth quartile (Q4) groups according to AI quartiles (32.42 ± 59.54% vs. 78.74 ± 198.50, *p* = 0.048; data are presented as mean ± SD) (Fig. 2A). However, no difference in the CAC volume was observed among the groups according to the HI quartile (Fig. 2B).

## Discussion

The association between OSA and CVD is well known through many studies. Hung et al. reported that OSA is a significant risk factor in patients with myocardial infarction as well as other well-known risk factors such as BMI, HTN, and smoking history^17^. Moreover, Shahar et al. reported that a modest increase in the odds ratio of CVD was observed in patients with severe OSA than in controls^18^. The pathophysiology of CVD in patients with OSA has been widely investigated and explained by increased daytime sympathetic activity, endothelial dysfunction, increases in inflammatory mediators, and increase in prothrombotic factors due to hypoxemia and apnea^19^. Therefore, we assumed that an assessment of the clinical association between OSA and the preclinical status of CVD is very important.

Several clinical studies have reported that CAC is a significant marker of subclinical atherosclerosis and is an important risk factor for CVD^1,5,20–23^. As mentioned above, the Agatston scoring method is the standard modality for assessing CAC^8^. The Agatston scoring method was first described by Agatston et al. in 1990 for the quantification of CAC using CT. To calculate the score, they up-weighted the high calcium density and multiplied the score and area^8^. Therefore, they regarded that both calcium density and volume are positively associated with the prediction of cardiovascular events. However, according to an article published later by Criqui et al.^24^, they suggested that a greater CAC density might be inversely related to upcoming CVD events. In this study, they used data from the MESA trial with 3398 participants and concluded that the CAC volume is independently associated with CVD risk. However, the CAC density was inversely and significantly associated with CVD, regardless of the CAC volume^24^. Thereafter, they confirmed their results with further assessment and concluded that the inverse association of CAC density and CVD risk is found at all levels of CAC volume^9^. Furthermore, the same authors reported factors that added to the atherosclerotic CVD risk score where the CAC volume and density provided the strongest prediction of CVD events^9^. Therefore, although our authors previously reported on the relationship between the CAC score calculated using the Agatston method and various factors related to OSA^6,7^, we believe that further evaluation using the CAC volume would be clinically meaningful, providing the basis for this study.

According to our present study results, we found that among various OSA-related parameters, the AI and minimal SaO_2_ were significantly associated with the CAC volume after adjustment for various confounders. We also found that the CAC score was significantly higher in the severe OSA group than in the HS group. Furthermore, it could be confirmed that this difference was more related to the AI than the HI. Therefore, based on the above results, we conclude that the AI and minimal SaO_2_ were independent factor associated with the CAC volume. Although it was not a study on the relationship between OSA and the CAC status, Hung et al. reported that only the AI was an independent predictor of MI among the various PSG parameters and suggested that the criterion of AI > 5 might be biologically important. In addition, they also reported that the AHI was predictive of MI before adjustment of various parameters, but it was no longer independently associated with MI after the AI was entered into a logistic model^17^. Therefore, they also concluded that the AI was important in predicting CVD events, and these results are very similar to ours. In fact, the AI was the primary disease-defining metric for OSA initially, but with obstructive hypopnea being widely recognized as having similar effects as obstructive apnea, physicians commonly use the AHI to evaluate the severity of OSA. However, apnea is a consequence of complete upper airway obstruction, and hypopnea is a result of partial obstruction^25^. Therefore, we considered that the possibility of apnea might have a greater adverse cardiovascular effect than hypopnea. In addition, previously we have been reported that the minimal SaO_2_ was significantly associated with the status of the CAC score^6^. Therefore, we regarded that minimal SaO_2_ is an important parameter of reflecting preclinical cardiovascular disease status as not only CAC score but also CAC volume.

Our study had several limitations. First, the participants of the present study were evaluated for CAC during routine health check-ups. They did not have any CVD-associated symptoms; thus, the national health insurance system did not cover the cost of the screening fee. Therefore, as the participants in this study were economically stable, this study might not reflect the general population and may have some selection bias. Second, the present study was a cross-sectional assessment with retrospective medical data review; thus, we could not evaluate the temporal relationship between the CAC volume and PSG parameters. Therefore, further studies on the serial evaluation of CAC volume changes according to various OSA-related parameters are warranted.

## Conclusion

In this study, we found that the AI and minimal SaO_2_ were significantly associated with the CAC volume after adjusting for confounders. In addition, when we assessed the CAC volume difference according to OSA severity, the mean CAC volume was significantly different between the HS and severe OSA groups. However, when we compared the CAC volume in the AI or HI quartile groups, the mean CAC volume was significantly different in Q4 versus Q1 of AI severity, but not in the HI quartile group. Therefore, among various OSA-related PSG parameters, the AI and minimal SaO_2_ were independently associated with the CAC volume and significantly related to upcoming CVD events in middle-aged male subjects.

## Data Availability

The data presented in this study are available on request from the corresponding author. The data are not publicly available due to privacy of patients.
